# Breast Cancer Exosome-like Microvesicles and Salivary Gland Cells Interplay Alters Salivary Gland Cell-Derived Exosome-like Microvesicles *In Vitro*

**DOI:** 10.1371/journal.pone.0033037

**Published:** 2012-03-20

**Authors:** Chang S. Lau, David T. W. Wong

**Affiliations:** 1 University of California Los Angeles School of Dentistry and Dental Research Institute, Los Angeles, California, United States of America; 2 University of California Los Angeles's Jonsson Comprehensive Cancer Center, Los Angeles, California, United States of America; 3 The Molecular Biology Institute at University of California Los Angeles, Los Angeles, California, United States of America; 4 University of California Los Angeles Division of Head and Neck Surgery/Otolaryngology, Los Angeles, California, United States of America; University of Kansas Medical Center, United States of America

## Abstract

Saliva is a useful biofluid for the early detection of disease, but how distal tumors communicate with the oral cavity and create disease-specific salivary biomarkers remains unclear. Using an *in vitro* breast cancer model, we demonstrated that breast cancer-derived exosome-like microvesicles are capable of interacting with salivary gland cells, altering the composition of their secreted exosome-like microvesicles. We found that the salivary gland cells secreted exosome-like microvesicles encapsulating both protein and mRNA. We also showed that the interaction with breast cancer-derived exosome-like microvesicles communicated and activated the transcriptional machinery of the salivary gland cells. Thus, the interaction altered the composition of the salivary gland cell-derived exosome-like microvesicles on both the transcriptomically and proteomically.

## Introduction

In an ongoing study, we use saliva, an accessible and non-invasive biofluid, for the early detection of diseases, such as Sjögren's syndrome or pancreatic, breast, and oral cancer [1]–[4]. Detecting the differential expression of salivary biomarkers between normal and diseased patients at both the mRNA and protein level allows us to detect specific diseases efficiently. We have shown that a combination of four RNA biomarkers (KRAS, MBD3L2, ACRV1, and DPM1) differentiates pancreatic cancer patients from non-cancer subjects (chronic pancreatitis and healthy controls), yielding a receiver operating characteristic (ROC) plot area under the curve value of 0.971 with 90.0% sensitivity and 95.0% specificity [4]. Although these translational and clinical findings provide an innovative breakthrough for the detection of systemic diseases, how distal systemic diseases mediate the presence of disease-indicating salivary biomarkers in the oral cavity remains unclear.

The present study demonstrates that interplay between salivary gland cells and tumor-derived exosome-like microvesicles induces *in vitro* changes in salivary gland cell-derived exosome-like microvesicles. Exosomes are cell-derived vesicles (30–100 nm in diameter) that stably reside in many body fluids, including blood, breast milk, urine, and saliva [5], [6], [7], [8]. Exosomes are formed by the inward budding of multi-vesicular bodies (MVBs), a component of the endocytic pathway [9], and consistently manufactured and secreted into the surrounding extracellular matrix and circulation through the fusion of MVBs with the plasma membrane [10], [11]. Due to their novelty, the physiological functions of exosomes have not yet been elucidated. Early studies first proposed that exosomes are secreted to discard membrane proteins [12]. However, more recent studies have shown that exosomes also contain antigens that are capable of triggering a biological immune response by activating T lymphocytes, natural killer cells, and dendritic cells [13]. Zitvogel et al. showed that dendritic cell-derived exosomes stimulate T-cell-mediated anti-tumor immune responses in mice [14]. Dendritic cell-derived exosomes were also found to express high levels of MHC class I and class-II peptides that trigger T-cell responses leading to tumor rejection [15]. Studies have also suggested that exosomes secreted by metastatic tumors provide interactions between the tumor front and distal host site, promoting tumor invasion by transporting RNA between cells, suppressing immune responses, and promoting angiogenesis [16].

These previous studies demonstrated that exosomes are durable for travel through body fluids and capable of intercellular communication. However, whether salivary gland cells are able to interact and take up tumor-derived exosome-like microvesicles has not been examined. Moreover, whether the interplay between tumor-derived exosome-like microvesicles and salivary gland cells alters salivary gland-derived exosome-like microvesicles is unknown. Because studies have shown that salivary gland cells readily secrete exosome-like microvesicles [17], we hypothesized that tumor-derived exosome-like microvesicles interact with salivary gland cells and alter the composition of their secreted exosome-like microvesicles in an *in vitro* setting. Using an *in vitro* breast cancer model, we investigated whether breast cancer-derived exosome-like microvesicles can communicate with salivary gland cells and if this interaction alters the exosome-like microvesicles released by salivary gland cells.

## Methods

### Reagents

The following reagents were used: Dulbecco's Modified Eagle Medium (DMEM, Invitrogen), fetal bovine serum (FBS, Cellgro), 50× penicillin/streptomycin (P/S, 5000 µg/ml, Cellgro), phosphate buffered saline (PBS, Invitrogen), Lipofectamine (Invitrogen), paraformaldehyde (Sigma), actinomycin D (ActD, Sigma), glutaraldehyde (Sigma), uranyl acetate (Sigma), simple stain solution (Invitrogen), CD63 antibody (Santa Cruz), β-actin antibody (Sigma), amylase antibody (Abcam), horseradish peroxidase-coupled secondary antibody (Invitrogen), RNase cocktail (Ambion), Triton X-100 (Sigma), and methanol (Sigma).

### Cell culture

Cells from the human metastatic mammary gland epithelial adenocarcinoma cell line MDA-MB-231 (231) [18] and human submandibular gland (HSG) cells [19] were cultured at 37°C with 5% CO_2_ in DMEM with 10% exosome-free FBS and 1× P/S. Exosomes were pre-cleared from the FBS via ultracentrifugation at 100,000 *g* for 2 hours and filtered using a 0.22 µm PVDF filter (Millipore). Cell count and viability were determined by the Vi-Cell viability analyzer (Beckman Couture).

### Isolation of exosome-like microvesicles

HSG and 231 cells were grown to 80% confluency and incubated in FBS-free DMEM for 48 hours. The culture supernatant was centrifuged at 300 *g* for 10 minutes to remove suspended cells. The cell pellet was discarded and the supernatant centrifuged at 2000 *g* for 10 minutes to remove dead cells, then 10,000 *g* for 30 minutes to remove cell debris. Next, the supernatant was centrifuged at 100,000 *g* for 70 minutes, the supernatant removed, the pellet washed with PBS and centrifuged at 100,000 *g* for 70 minutes, then filtered using a 0.22 µm PVDF syringe filter (Millipore), resulting in purified exosome-like microvesicles.

### Electron microscopy

Isolated exosome-like microvesicles were re-suspended and fixed with 2% PFA. The microvesicles were then deposited onto charged carbon-coated grids (Ted Pella) followed by post-fixation using 1% gluteraldehyde and washed three times with distilled water. Samples were then contrasted with 2% uranyl–acetate solution and examined with an electron microscope. Films were scanned, gamma adjusted, and assembled using Adobe Photoshop CS, Adobe Illustrator CS, and Image J.

### SDS-PAGE and protein staining

Exosome-like microvesicles and cell lysates were re-suspended in Laemmli Sample Buffer (62.5 mM Tris-HCl pH 6.8, 2% wt/vol sodium dodecyl sulfate, 50 mM dithiothreitol, 0.01% wt/vol) and analyzed by sodium dodecyl sulfate polyacrylamide gel electrophoresis (SDS-PAGE) followed by staining according to the manufacturer's instructions.

### Western blotting

The membrane was blocked with 1% milk solution and incubated with CD63, β-actin, or amylase antibody, followed by incubation with the appropriate horseradish peroxidase-coupled secondary antibody. The proteins were detected using the Amersham ECL Western Blotting Detection System (GE Healthcare).

### Exosomal RNA extraction and analysis

HSG and 231-derived exosome-like microvesicles were treated with RNase cocktail (final concentration 100 U/ml) with or without 3% Triton X-100 at room temperature for 20 minutes. Exosomal RNA was extracted from the lysed microvesicles using the RNeasy Mini Kit (Qiagen) according to the manufacturer's instructions. The isolated RNA was analyzed using the RNA 6000 Pico Kit and Bio-analyzer (Agilent).

### Communication assays

231-derived exosome-like microvesicles were introduced to serum-starved HSG cells in DMEM for 12 hours (at 37°C, 5% CO_2_), then subsequently washed three times with PBS, trypsinized, detached, and centrifuged to isolate HSG cells for RNA extraction. Cell count and viability were determined using the Vi-Cell viability analyzer. As a control, lysed exosome-like microvesicles (3% Triton X-100 treated) were introduced to HSG cells. HSG cells were also treated with ActD (0.2 µg/ml) prior to treatment with 231-derived exosome-like microvesicles for 10 minutes. HSG RNA was extracted using the RNeasy Kit according to the manufacturer's instructions. RNA concentrations were determined using a Nanodrop 3000 (Thermo) and RNA quality analyzed by the RNA 6000 Nano Kit (Agilent). RNA concentrations were normalized to cell count. For the microarray analysis of HSG exosome-like microvesicles, serum-starved HSG were treated with 231-derived exosome-like microvesicles for 12 hours, washed three times with PBS, and cultured in serum-free DMEM at 37°C and 5% CO_2_ for 48 hours. For control purposes, lysed exosome-like microvesicles were introduced to HSG cells. Exosome-like microvesicles were isolated from the HSG culture media by ultracentrifugation. The RNeasy Kit was utilized to extract RNA from the isolated HSG-derived exosome-like microvesicles, and the RNA was amplified/purified using the RiboAMP RNA Amplification Kit (Molecular Devices). The cDNA was transcribed and biotinylated using the GeneChip Expression 3′-Amplication Kit (Affymetrix). GeneChip (HGU-133 Plus 2.0) hybridization and scanning were performed at the UCLA Gonda Microarray Core Facility. A heat map of the microarray results was generated by JMP 9.0.2 (http://www.jmp.com). For exosomal protein analysis, HSG-derived exosome-like microvesicles (isolated after treatment with 231-derived exosome-like microvesicles or lysed exosome-like microvesicles) were diluted in 30 µl of PBS and sent to Applied Biomics for 2-dimensional difference gel electrophoresis (2D-DIGE). An equal amount of protein sample was labeled with Cy2 as an internal standard. Experimental and control samples were labeled with Cy3 or Cy5, and three gels were run for comparative analysis of three separate preparations. The three gels were matched by the biological variance analysis module of DeCyder 6.5. Spot volume was normalized within the gel, and the abundance of each spot was normalized against the internal Cy2 standard so that spots could be compared across gels. The ratio of experimental (Exo) to control (Lys Exo) was calculated for each spot, and the average ratio and *P*-values (Student *t* test and one-way ANOVA) from three replicate samples were calculated using DeCyder 6.5 software.

### U133 Plus 2.0 Array data analysis and gene ranking

Arrays were analyzed using R 2.7.0 (http://www.r-project.org). The probe logarithmic intensity error estimation (PLIER) expression measures were computed after background correction and quantile normalization for each microarray data set. Probe set-level quantile normalization was performed across all samples to make the effect sizes similar among all data sets. For every probe set, the 2-sample *t*-test was applied to identify differential expression between samples treated with 231-derived exosome-like microvesicles or lysed 231-derived exosome-like microvesicles. After obtaining the estimates and *P*-values for each probe set, we corrected the *P*-values for the false discovery rate. A score was then generated based on the corrected *P*-values and differential expression levels.

### Exosome labeling and transfer assay

231-derived exosome-like microvesicles were isolated and labeled using the PKH26 Red (543 nm excitation) Fluorescent Cell Linker Mini Kit according to the manufacturer's directions. The labeled exosome-like microvesicles were introduced to HSG cells cultured in DMEM with 10% exosome-free FBS and P/S for 1 hour at 37°C and 5% CO_2_. For control purposes, lysed labeled exosome-like microvesicles and PKH only were introduced to HSG cells. If the PKH molecule is not encapsulated within the exosome-like microvesicles, the labeling reaction will be stopped by the serum proteins in the cell medium [20]. Next, HSG cells were fixed with 100% methanol, washed with PBS, and imaged at 400× (40× objective and 0.50 numerical aperture) at room temperature using a Leica DM1L inverted light microscope attached to a Prior Lumen 200 Argon light-box and Zeiss Axiocam MRm camera. The images were acquired using Axiovision Rel. 4.8 software. For quantification, HSG were trypsinized, centrifuged, re-suspended in PBS, and analyzed via FACS at the UCLA Flow Cytometry Core. The images were gamma adjusted and assembled using Adobe Photoshop CS, Adobe Illustrator CS, and Image J.

### Statistical analysis

All graphs were made and statistical analyses performed using R 2.7.0, GraphPad Prism, or Microsoft Excel 2008. All experiments were performed a minimum of three times. One-way ANOVA and 2-sample *t*-tests were used to determine significance (*P*-values<0.05). Data are expressed as mean ± SEM.

## Results

### HSG and 231-derived exosome-like microvesicles contain proteins and mRNA

Electron microscopy (58 K and 100 K magnification) images taken of isolates from the culture media of HSG and 231 cells showed that they secreted exosome-like microvesicles (Figure S1A). The isolated exosome-like microvesicles from both cell lines were between 30–100 nm in size and round with cup-like concavity. SDS-PAGE analysis found that both HSG and 231 exosome-like microvesicles contained proteins distinct from their parental cell lysates, and immunoblot detected exosomal marker CD63 in exosome-like microvesicles from both cell lines (Figure S1B and C). In addition to the related band at ∼40 kDa, both HSG and 231-derived exosome-like microvesicles expressed CD63 at a distinctly higher molecular weight compared to the CD63 found in their parental cells. To verify that HSG and 231-derived exosome-like microvesicles contained mRNA, they were treated with RNase with or without 3% Triton and then analyzed (Figure S1D and E). For both cell lines, degradation of exosomal mRNA was observed only after the exosome-like microvesicles were lysed with 3% Triton, indicating that both HSG and 231-derived exosome-like microvesicles encapsulated mRNA.

### PKH-labeled 231-derived exosome-like microvesicles can label human salivary gland cells in the presence of serum proteins

**Figure 1:**
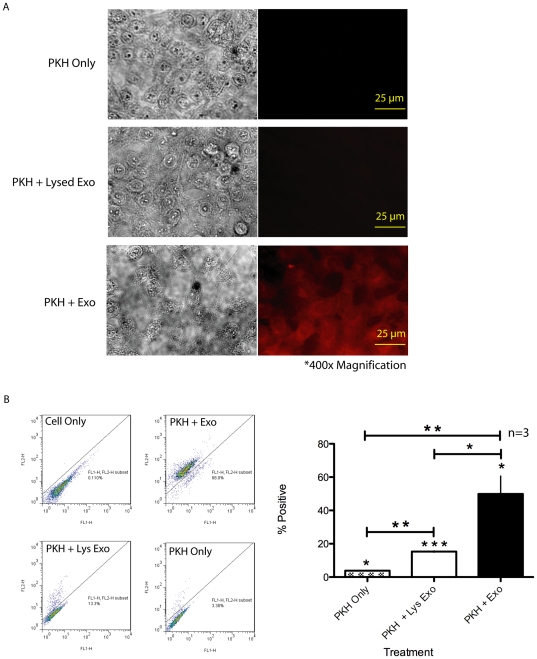
PKH-labeled 231-derived exosome-like microvesicles can label human salivary gland cells in the presence of serum. (A) Microscopy (scale bar = 25 µm) and (B) FACS results showed that HSG cells were labeled after treatment for 1 hour with PKH-labeled 231-derived exosome-like microvesicles, and minimally labeled when treated only with PKH dye or lysed microvesicles. * *P*<0.05, ** *P*<0.01, and *** *P*<0.001; n = 3. All experiments were independently performed a minimum of three times.

231-derived exosome-like microvesicles were labeled with red fluorescent lipid linker PKH and then introduced to HSG cells by adding the labeled exosome-like microvesicles to the conditioned media with 10% exosome-free FBS. We observed that the PKH compound from the labeled 231-derived exosome-like microvesicles transferred to the HSG cells, protecting the PKH26 molecule from being quenched by the serum (Figure 1). Microscopy showed that HSG cells were labeled only upon treatment with PKH-containing 231-derived exosome-like microvesicles, not when treated with PKH only or PKH-labeled exosome-like microvesicles lysed with 3% Triton (Figure 1A). FACS analysis confirmed the significant microscopy results; 49.89±10.77% of total HSG cells were labeled upon treatment with PKH-labeled 231-derived exosome-like microvesicles. The 2-sample *t*-test revealed a significant decrease in the percentage of total HSG cells labeled upon treatment with PKH only or lysed PKH-labeled exosome-like microvesicles, as only 3.77±0.23% and 15.32±0.39% of total HSG cells were labeled, respectively (Figure 1B).

### Up-regulation of total RNA in HSG cells induced by 231-derived exosome-like microvesicles

**Figure 2:**
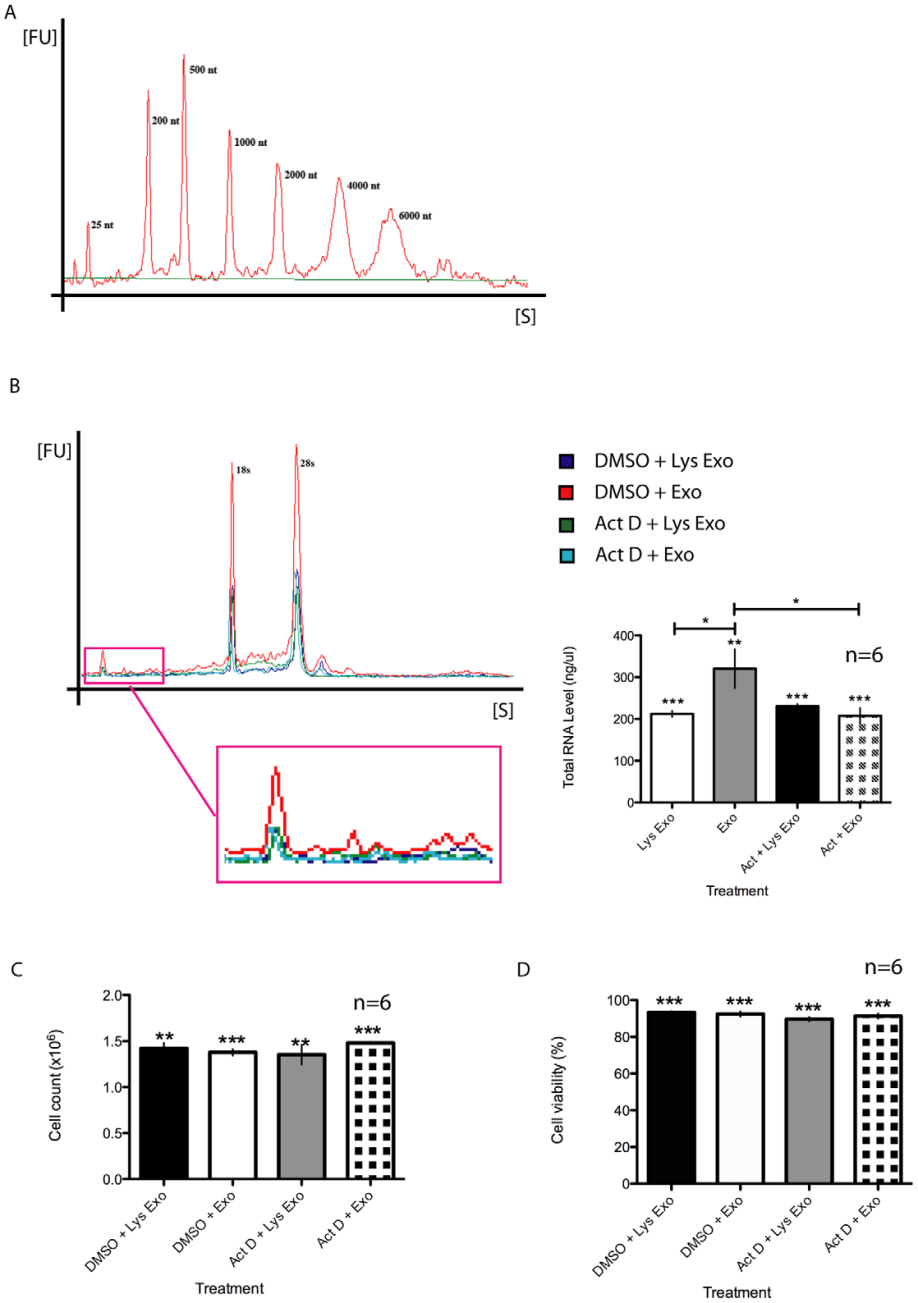
Up-regulation of HSG RNA induced by 231-derived exosome-like microvesicles. A) Nano Ladder. (B) Basal RNA level in serum-starved HSG cells, along with 18 s and 28 s ribosomal RNA peaks. Increased total RNA levels were observed after a 12-hour treatment with 231-derived exosome-like microvesicles (Exo) compared to lysed 231-derived exosome-like microvesicles (control, Lys Exo). Transcription inhibition by actinomycin D (ActD) diminished the increase in RNA levels induced by 231-derived exosome-like microvesicles, suggesting that transcription is activated by 231-derived exosome-like microvesicles. (C) Cell count and (D) cell viability were not affected by the treatments. * *P*<0.05, ** *P*<0.01, and *** *P*<0.001; n = 6. All experiments were independently performed a minimum of three times.

Total RNA was up-regulated in HSG cells after treatment with 231-derived exosome-like microvesicles compared to samples treated with lysed 231-derived exosome-like microvesicles (Figure 2). Analysis of total cellular RNA from serum-starved HSG cells detected two ribosomal RNA (rRNA) peaks and a basal level of cellular RNA. After 12 hours of incubation with 231-derived exosome-like microvesicles, 2-sample *t*-test indicated significantly more total RNA in HSG cells treated with 231-derived exosome-like microvesicles compared to control (206.7±10.37 ng/µl vs. 349.3±96.06 ng/µl, Figure 2B). This increase in total RNA significantly diminished upon pre-treatment of the HSG cells with transcription inhibitor ActD (Figure 2B). HSG cell count and viability were not affected and significantly different upon treatment with exosome-like microvesicles, lysed exosome-like microvesicles, or ActD (Figure 2C and D).

### HSG-derived exosomal protein content was altered by 231-derived exosome-like microvesicles

**Figure 3:**
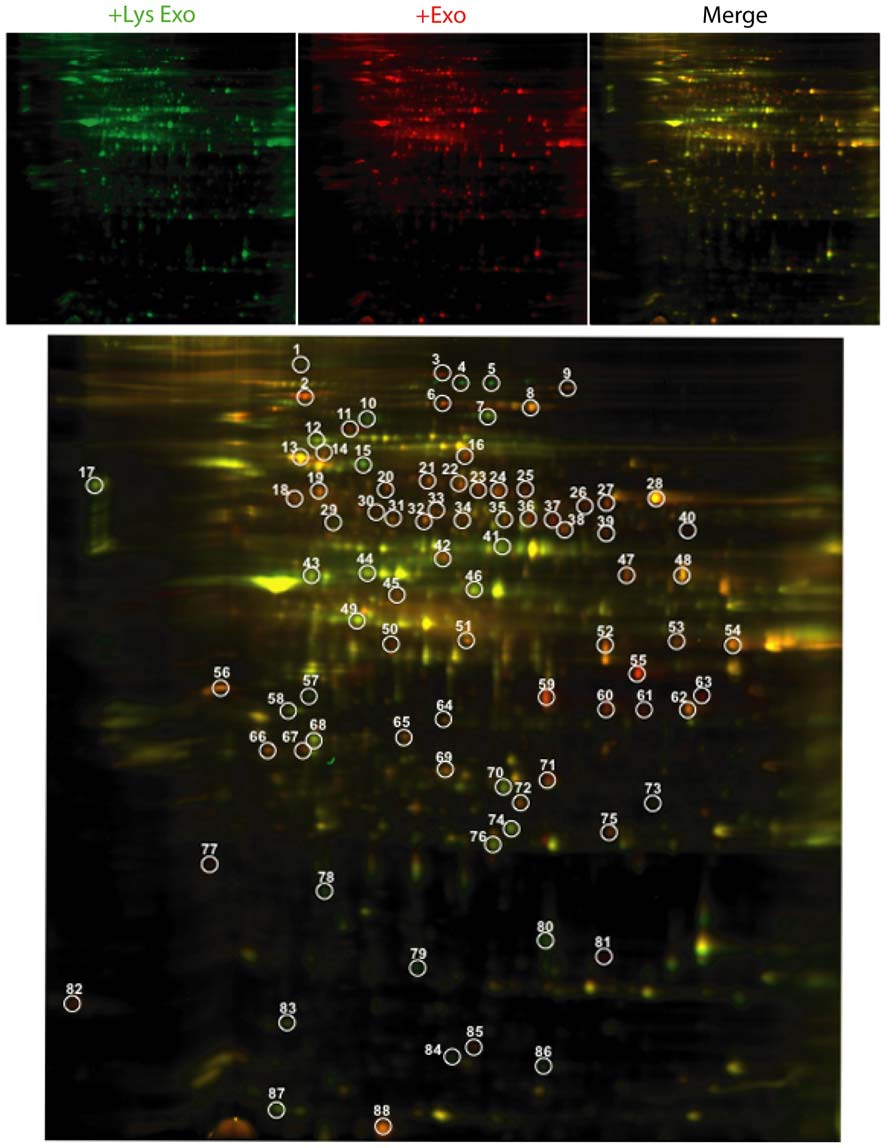
HSG-derived exosomal protein content was altered by 231-derived exosome-like microvesicles. 2D-DIGE identified 88 spots (circled) differing by 1.5-fold or more were observed compared to HSG-derived exosomal proteins treated with lysed 231-derived exosome-like microvesicles after treatment with intact 231-derived exosome-like microvesicles (n = 1).

231-derived exosome-like microvesicles were introduced to serum-starved HSG cells for 12 hours and HSG-derived exosome-like microvesicles isolated from the media 48 hours later. The microvesicles were lysed and extracted proteins analyzed via 2D-DIGE (Figure 3). Microvesicles isolated from HSG cells treated with 231-derived exosome-like microvesicles contained 88 proteins that were present at levels at least1.5-fold higher than in the control sample. The exosomal proteins that were differentially packaged ranged in size from larger than 150 KDa to smaller than 15 KDa, and in pH from lower than 4.0 to higher than 8.0.

### Interplay between 231-derived exosome-like microvesicles altered the composition of HSG cell exosomal mRNA

**Figure 4:**
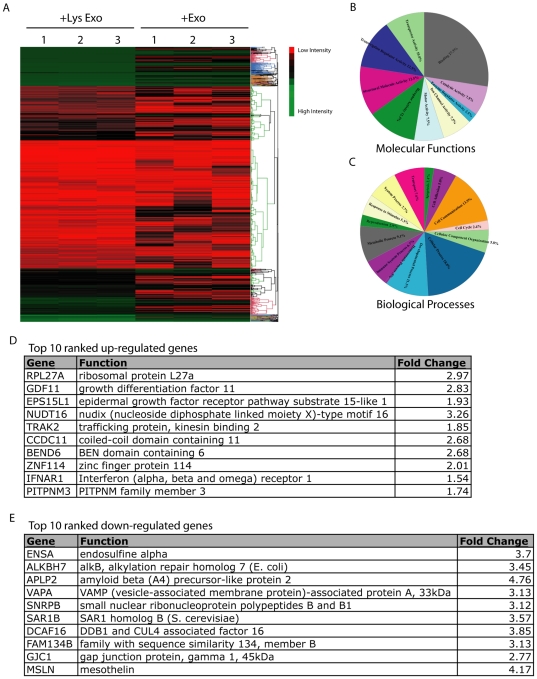
Interplay between 231-derived exosome-like microvesicles altered the HSG exosomal mRNA composition. (A) Heat map of microarray analysis results for mRNA transcripts from exosome-like microvesicles isolated from HSG cells treated with 231-derived exosome-like microvesicles or lysed 231-derived exosome-like microvesicles (control). (B) Ontological analysis of the 66 mRNA transcripts distinct to exosome-like microvesicles derived from HSG cells that interacted with 231-derived exosome-like microvesicles implicated in various molecular functions and (C) biological processes. (D, E) Array analysis using R 2.7.0 revealed the top 10 up- or down-regulated HSG-derived exosomal mRNA transcripts after treatment with 231-derived exosome-like microvesicles with respect to control. The results were generated via three independent trials.

The comparison of exosomal RNA isolated from HSG cells treated with 231-derived exosome-like microvesicles or lysed exosome-like microvesicles (control) revealed 66 significant mRNAs specific to HSG-derived exosome-like microvesicles treated with 231-derived exosome-like microvesicles (Table S1). The heat map of the microarray showed differential mRNA expression patterns between HSG-derived exosome-like microvesicles treated with 231-derived exosome-like microvesicles and the control (Figure 4A). A gene ontology-based analysis (http://www.pantherdb.org) implicated the 66 significant mRNAs distinct to HSG-derived exosome-like microvesicles treated with 231-derived exosome-like microvesicles in various cellular and physiological processes, ranging from cell cycle to metabolism (Figure 4B and C). Furthermore, the composition of HSG-derived exosomal mRNA was affected by post-treatment with 231-derived exosome-like microvesicles. Figure 4D and E list the top 10 up- or down-regulated HSG-derived exosomal mRNA after treatment with 231-derived exosome-like microvesicles with respect to control.

## Discussion

Saliva is an effective, non-invasive biofluid for the detection of various diseases, such as pancreatic, oral, and breast cancer [1]. In this study, we demonstrated that the interplay between 231-derived exosome-like microvesicles and HSG cell altered HSG-derived exosome-like microvesicles. We showed that both HSG and 231 cells are capable of secreting exosome-like microvesicles encapsulating protein and mRNA. In addition, we observed that the PKH-labeled 231-derived exosome-like microvesicles were able to label HSG cells in the presence of serum. Moreover, the interplay between 231-derived exosomes and HSG cells activated the HSG cell transcriptional machinery, inducing an up-regulation of total cellular RNA. We also discovered that interactions between HSG cells and 231-derived exosome-like microvesicles altered the HSG-derived exosome-like microvesicles both proteomically and transcriptomically.

The examination of isolates from the culture media of 231 and HSG cells showed that both cell lines secreted exosome-like microvesicles in abundance. Isolates from both 231 and HSG cells were identified as exosome-like microvesicles due to their size (30–100 nm) and morphology (round with cuplike concavity). In addition, the exosomal marker tetraspanin CD63 was found in both 231- and HSG-derived exosome-like microvesicles. In addition to the expected band for CD63 at 40 KDa, a 55 KDa version of CD63 was detected in the exosome-like microvesicle lysates from both cell lines. The size differences between exosome-like microvesicle and cell lysate CD63 may be due to the glycosylation-prone nature of this membrane protein [21]. Moreover, amylase protein was found in HSG-derived exosome-like microvesicles and the cell lysates (Figure S3), indicating that HSG cells have acinar cell-like characteristics. We also observed that HSG readily secreted exosome-like microvesicles encapsulating both mRNA and proteins, suggesting that these HSG cells are capable of secreting biomarker-enriched exosome-like microvesicles. These results are consistent with the findings of Gonzales-Begne et al. [7], who found 914 total parotid gland-derived exosomal proteins, and with our previous work in which we found that salivary exosome-like microvesicles contain proteins and functional mRNA.

The precise mechanism underlying why disease-specific salivary biomarkers are present in the saliva remains unclear. Studies have shown that exosomes can stably reside in body fluids, including urine, blood, milk, and saliva [5]–[8]. Thus, we believe exosomes provide a credible means for intercellular communication. Because salivary exosomes are released into the saliva via ductal or acinar cells [22], salivary gland cells may interact with circulating tumor exosomes in the vasculature and reflect this interaction in the exosomes secreted into the saliva.

We found that PKH-labeled 231-derived exosome-like microvesicles were capable not only of protecting the PKH molecule from quenching by serum, but also labeling HSG cells. Thus, even though we do not show the transference of proteins or mRNA, this result suggests that 231-derived exosome-like microvesicles are capable of transferring their exosomal materials to HSG cells. Because we observed that only approximately half of the HSG cell populations were labeled, the heterogeneity of the cell line itself may contribute to this variation in exosome uptake. Thus, to examine whether the HSG cell population has variations in 231-derived exosome-like microvesicle uptake, we introduced PKH-labeled 231-derived exosome-like microvesicles to HSG cells at various dilutions (1∶1, 1∶2, 1∶4, 1∶8). Using fluorescence activated cell sorting (FACS) we observed a decrease in HSG cell labeling as the concentration of the input PKH-labeled 231-derived exosome-like microvesicles decreased (Figure S2). This finding indicates that the concentration of the labeled 231-derived exosome-like microvesicles that is introduced and the random encounter and uptake of these microvesicles by the HSG cells results in the labeling of ∼50% of the cells, rather than the heterogeneity of the HSG cell population. We also observed that the interactions between 231-derived exosome-like microvesicles and HSG cells induced an overall up-regulation of their total RNA levels at the transcriptional level. However, we were unable to detect any obvious phenotypic alterations to the HSG cells. Thus, while the rationale is unclear and beyond the scope of this study, we reason that there could be multitude of reasons that are at the molecular and biological levels that may be worthwhile to pursue for future studies.

The literature suggests several possible mechanisms by which exosomes can enter a cell, transfer material, and activate transcription. First, exosomes are capable of fusing with cell membranes and directly entering the cytoplasm [13]. Alternatively, exosomes can enter a cell passively via clathrin and receptor-mediated processes [13]. Studies have identified micro-RNA (miRNA) and transcription factors in exosomes of various origins [23]. Thus, exosomes may transfer their contents to induce transcription. Exosomes have also been proposed to interact with a target cell in a juxtacrine fashion, by ectodomain cleavage leading to exosomal fragments acting as ligands, or direct fusion with the target cell [9]. Juxtacrine communication and ectodomain cleavage are thought to allow exosomal proteins to interact with the target cell receptors, leading to cell activation.

Here, we showed that the interplay between 231-derived exosome-like microvesicles and HSG cells *in vitro* alters the HSG-derived exosome-like microvesicles proteomically. Several models have been proposed in regards to exosome uptake and protein trafficking that may be useful for future investigations into their mechanism. Due to the heterogeneity of exosomal proteins, which range from transmembrane proteins to chaperones [9], exosomal protein packaging may be both endosomal sorting complex required for transport (ESCRT)-dependent and/or independent depending on cellular localization [24]. Based on the proposed models of protein sorting to intra-luminal vesicles (ILVs) of the MVBs, exosomes internalized into cells via clathrin-mediated endocytosis are postulated to enter the endosomes for sorting, and are either sent to the lysosomes for degradation or re-packaged into the host's exosomes in an ESCRT-dependent manner [25]. Alternatively, exosomes may directly fuse with cellular membranes and unload their cargo proteins into the target's cytosol [26]. Thus, the non-specific uptake of cytosolic proteins during inward budding processes and/or transient association between cytosolic proteins and transmembrane proteins may possibly lead to sequestration of the newly acquired proteins into the re-packaged exosomes.

Translation of exosomal mRNA can also play a role in the target cell's exosomal protein composition. Exosomes may encapsulate transferable and functionally active mRNA, and exosomal mRNA newly transferred into the target cell's cytosol may be translated by free-floating ribosomes [27]. Thus, newly translated cytosolic proteins may be sequestered into the target cell's ILVs of the MVBs during inward budding processes, and consequently packaged and released in exosomes.

In addition to proteomic changes, microarray analysis revealed that the interplay between 231-derived exosome-like microvesicles and HSG cells altered the mRNA composition of HSG-derived exosome-like microvesicles. The literature suggests that interactions between exosomal ligands and cellular receptors can induce cellular activation, leading to nascent mRNA transcripts [9]. Therefore, the direct fusion of exosomes with the target cell can lead to unloading of exosomal mRNA into the cytosol where basal inward budding processes occur and trigger the sequestration of novel exosomal mRNA into newly synthesized exosomes.

Here, we showed that the interplay between 231-derived exosome-like microvesicles alters HSG-derived exosome-like microvesicles both transcriptomically and proteomically. However, because this is an *in vitro* study, we are unable to make the assumption that breast cancer cell-derived exosomes induce breast cancer-specific biomarkers released from the salivary glands. Instead, based on our observations, we can suggest that within an *in vivo* setting, if breast cancer-derived exosome-like microvesicles were to reach the salivary glands, and if breast cancer-derived exosome-like microvesicles are internalized by the salivary gland cells, the composition of released salivary gland-derived exosome-like microvesicles will change both transcriptomically and proteomically.

The mechanism underlying the alteration of HSG-derived exosomal composition is unknown. However, previous findings in regards to exosomal biogenesis and cellular cargo trafficking provide us a solid foundation for further investigation. Examining how acquired cancer-derived exosomal contents are packaged in salivary gland cell-derived exosomes will be crucial for decoding the mechanism underlying the existence of salivary biomarkers. Furthermore, understanding how cancer-derived exosomes enter the salivary gland cells will provide us with a clue as to whether salivary biomarkers are directly derived from the disease source (i.e. exosomes enter the salivary gland cells, are packaged into MVBs, and released) or whether secondary messengers are involved (i.e. exosomes unload mRNA into salivary gland cells, mRNA is translated by free cytosolic ribosomes, and newly synthesized peptides are sequestered and released).

## Supporting Information

Figure S1 **HSG and 231 cells secreted exosome-like microvesicles containing proteins and mRNA.** (A) HSG and 231-derived exosome-like microvesicles were isolated from culture media and visualized by electron microscopy (scale bar = 100 nm). (B) SDS-PAGE of exosomal lysates from HSG and 231 cells revealed distinct protein composition compared to their parental cell lysates. (C) Both 231- and HSG-derived exosome-like microvesicles contained the exosomal marker CD63. (D) Agilent Bio-Analyzer Pico analysis shows that mRNA is encapsulated in exosome-like microvesicles derived from HSG and (E) 231 cells. When treated with 3% Triton to lyse the exosome-like microvesicles, RNase was able to readily degrade the exosomal mRNA. All experiments were independently performed a minimum of three times.
(TIF)

Figure S2 **FACS analysis of PKH labeling of HSG cells by 231-derived exosome-like microvesicles at various dilutions.** FACS analysis demonstrated that the labeling of HSG cells by PKH-labeled 231-derived exosome-like microvesicles decreases as the input concentration of PKH-labeled 231-derived exosome-like microvesicles decreases.
(TIF)

Figure S3 **Amylase protein is found in HSG cells and HSG-derived exosome-like microvesicles.** Western blot revealed that amylase protein is produced in HSG cells and also secreted in HSG-derived exosome-like microvesicles. Amylase protein found in HSG cell lysates had a molecular weight of ∼56 KDa, whereas the amylase protein found in HSG-derived exosome-like microvesicles was ∼56 KDa and ∼62 KDa due to differential glycosylation. Amylase protein was not found in 231 cell lysates.
(TIF)

Table S1 **Interplay between 231-derived exosome-like microvesicles and HSG cells altered the composition of exosomal mRNA in HSG cells.** We found 66 significant mRNA transcripts specific to HSG-derived exosome-like microvesicles treated with 231-derived exosome-like microvesicles after three independent trials.
(PDF)
