# Supplementary figures and images for: Breast Cancer Exosome-like Microvesicles and Salivary Gland Cells Interplay Alters Salivary Gland Cell-Derived Exosome-like Microvesicles In Vitro
Source: PLoS One. 2012 Mar 20;7(3):e33037. doi: 10.1371/journal.pone.0033037 (PMC3308964; doi:10.1371/journal.pone.0033037)

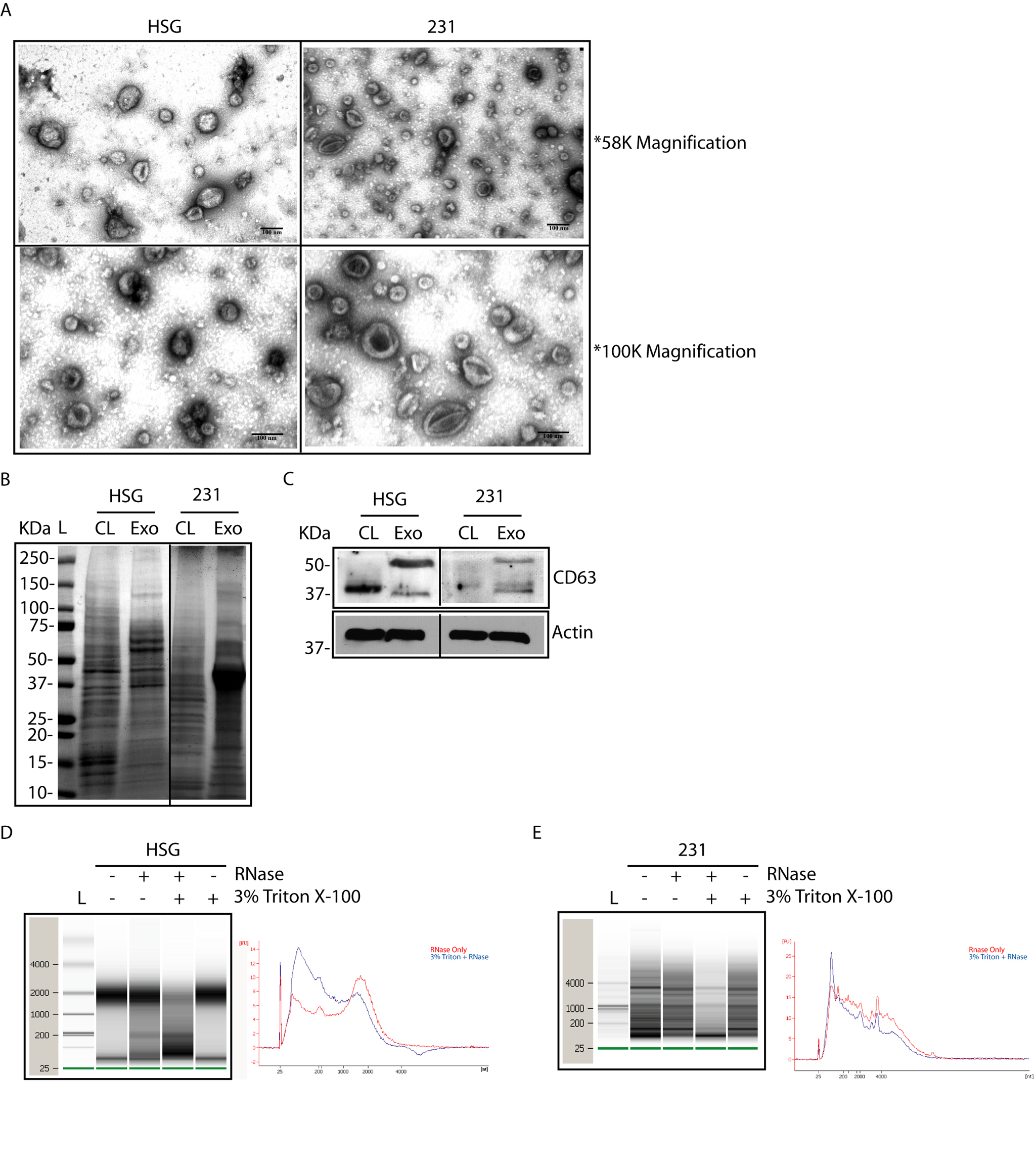

Supplement: Figure S1 — HSG and 231 cells secreted exosome-like microvesicles containing proteins and mRNA. (A) HSG and 231-derived exosome-like microvesicles were isolated from culture media and visualized by electron microscopy (scale bar = 100 nm). (B) SDS-PAGE of exosomal lysates from HSG and 231 cells revealed distinct protein composition compared to their parental cell lysates. (C) Both 231- and HSG-derived exosome-like microvesicles contained the exosomal marker CD63. (D) Agilent Bio-Analyzer Pico analysis shows that mRNA is encapsulated in exosome-like microvesicles derived from HSG and (E) 231 cells. When treated with 3% Triton to lyse the exosome-like microvesicles, RNase was able to readily degrade the exosomal mRNA. All experiments were independently performed a minimum of three times. (TIF) [file pone.0033037.s001.tif]

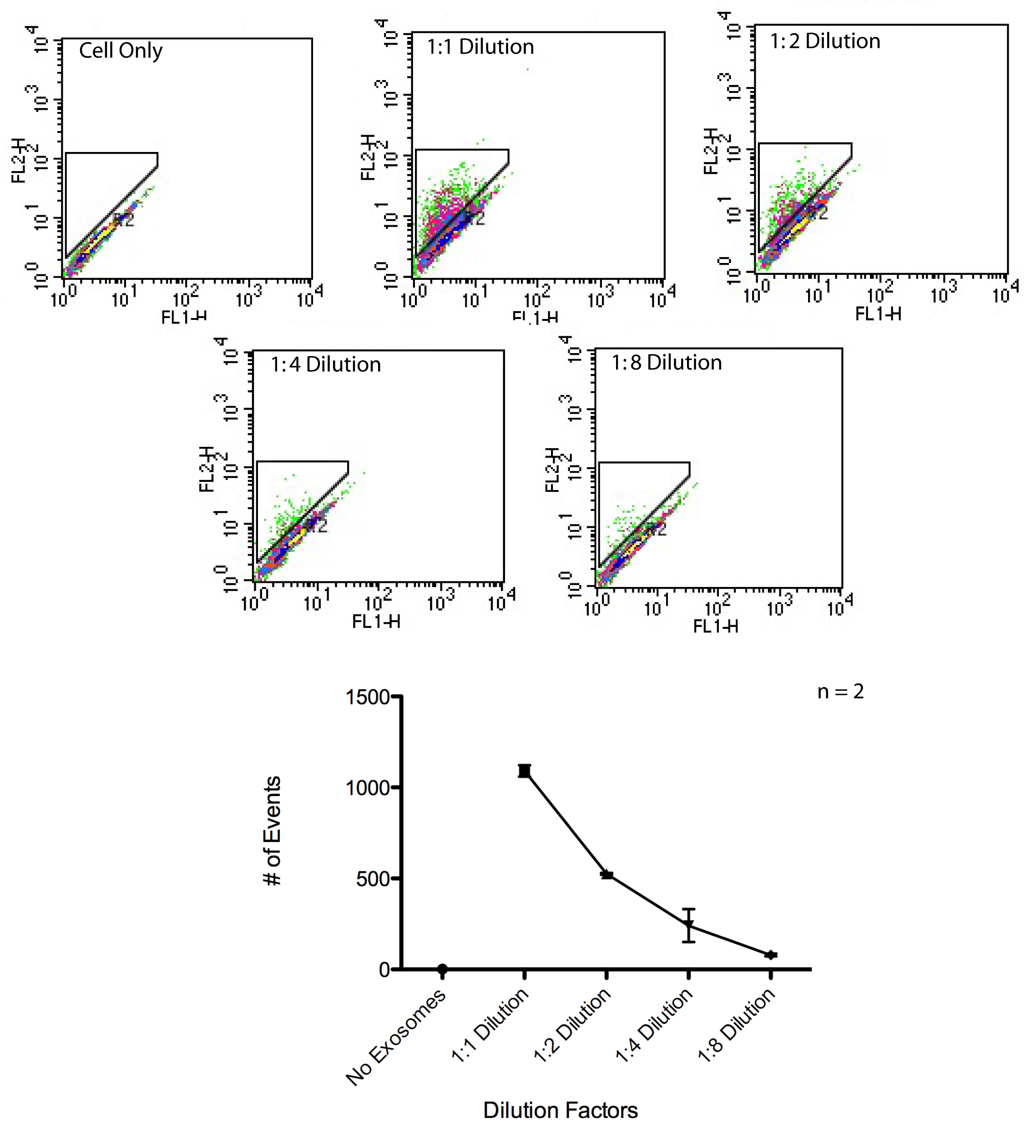

Supplement: Figure S2 — FACS analysis of PKH labeling of HSG cells by 231-derived exosome-like microvesicles at various dilutions. FACS analysis demonstrated that the labeling of HSG cells by PKH-labeled 231-derived exosome-like microvesicles decreases as the input concentration of PKH-labeled 231-derived exosome-like microvesicles decreases. (TIF) [file pone.0033037.s002.tif]

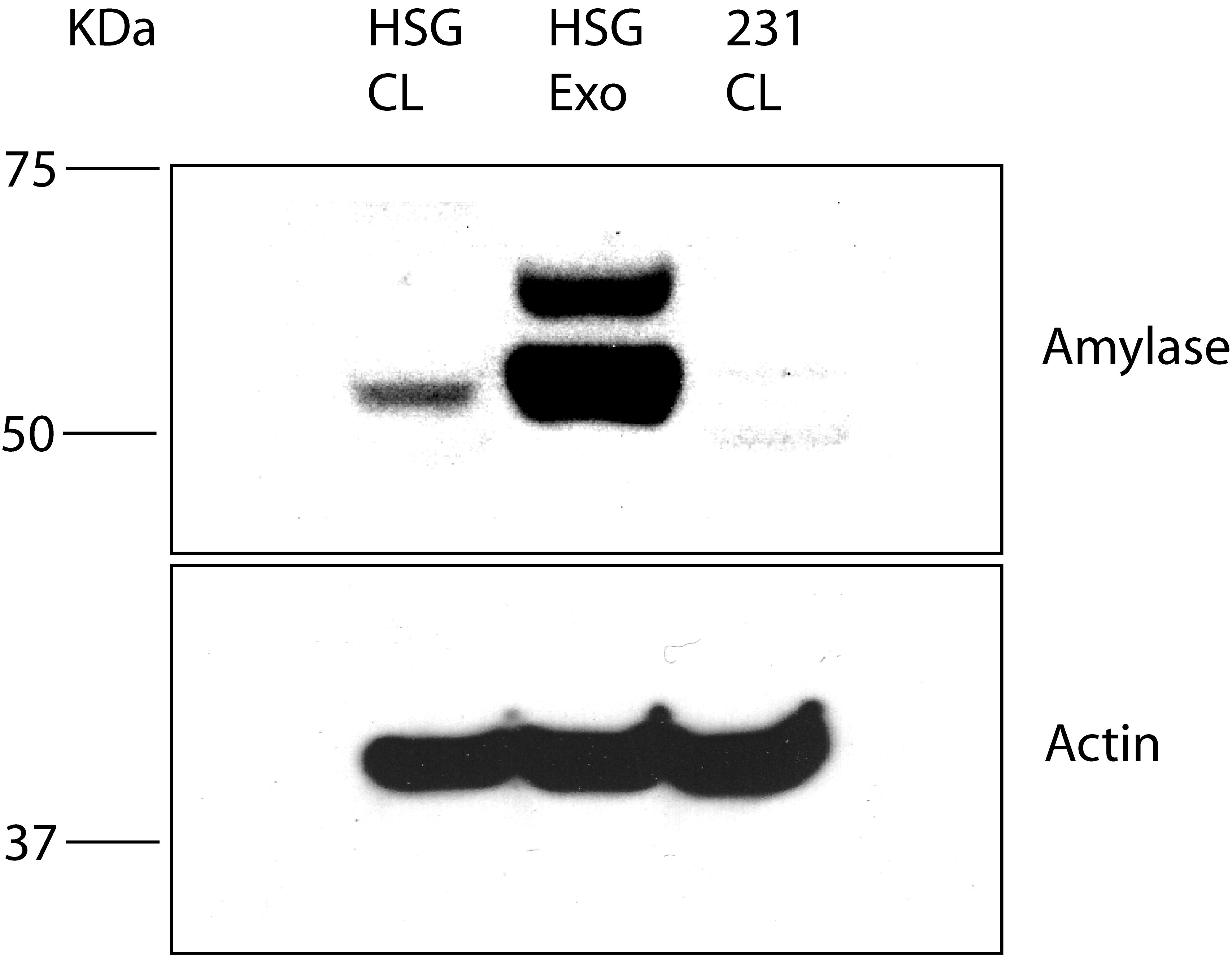

Supplement: Figure S3 — Amylase protein is found in HSG cells and HSG-derived exosome-like microvesicles. Western blot revealed that amylase protein is produced in HSG cells and also secreted in HSG-derived exosome-like microvesicles. Amylase protein found in HSG cell lysates had a molecular weight of ∼56 KDa, whereas the amylase protein found in HSG-derived exosome-like microvesicles was ∼56 KDa and ∼62 KDa due to differential glycosylation. Amylase protein was not found in 231 cell lysates. (TIF) [file pone.0033037.s003.tif]
