# Supplementary material for: Breast Cancer Exosome-like Microvesicles and Salivary Gland Cells Interplay Alters Salivary Gland Cell-Derived Exosome-like Microvesicles In Vitro
Source: PLoS One. 2012 Mar 20;7(3):e33037. doi: 10.1371/journal.pone.0033037 (PMC3308964; doi:10.1371/journal.pone.0033037)
Supplement: Table S1 — Interplay between 231-derived exosome-like microvesicles and HSG cells altered the composition of exosomal mRNA in HSG cells. We found 66 significant mRNA transcripts specific to HSG-derived exosome-like microvesicles treated with 231-derived exosome-like microvesicles after three independent trials. (PDF) [file pone.0033037.s004.pdf]

Gene	Function
A2BP1	ataxin 2-binding protein 1
ABCC13	ATP-binding cassette, sub-family C (CFTR/MRP), member 13, pseudogene
ADAM12	ADAM metallopeptidase domain 12
ARHGAP10	Rho GTPase activating protein 10
ATXN7L1	ataxin 7-like 1
B9D2	B9 protein domain 2
C1QTNF5 /// MFRP	C1q and tumor necrosis factor related protein 5 /// membrane frizzled-related protein
CABP4	calcium binding protein 4
CACNA2D3	calcium channel, voltage-dependent, alpha 2/delta subunit 3
CACNB2	calcium channel, voltage-dependent, beta 2 subunit
CADM1	cell adhesion molecule 1
CDH11	Cadherin 11, type 2, OB-cadherin (osteoblast)
CELF2	CUGBP, Elav-like family member 2
CRB1	crumbs homolog 1 (Drosophila)
CTDSP1	CTD (carboxy-terminal domain, RNA polymerase II, polypeptide A) small phosphatase 1
CXorf48	chromosome X open reading frame 48
ELAVL2	ELAV (embryonic lethal, abnormal vision, Drosophila)-like 2 (Hu antigen B)
ELSPBP1	epididymal sperm binding protein 1
EPS8L1	EPS8-like 1
EXOC3L2	exocyst complex component 3-like 2
FAT3	FAT tumor suppressor homolog 3 (Drosophila)
GABRB1	gamma-aminobutyric acid (GABA) A receptor, beta 1
GABRB2	gamma-aminobutyric acid (GABA) A receptor, beta 2
GLP1R	glucagon-like peptide 1 receptor
GPR68	G protein-coupled receptor 68
HIST1H3H	histone cluster 1, H3h
HIST2H2AA3	histone cluster 2, H2aa3
HTRA4	HtrA serine peptidase 4
IFNA1 /// IFNA13	interferon, alpha 1 /// interferon, alpha 13
IL1RAPL1	interleukin 1 receptor accessory protein-like 1
IRF8	interferon regulatory factor 8
KCNJ15	potassium inwardly-rectifying channel, subfamily J, member 15
KIF5A	Kinesin family member 5A
KIF5C	kinesin family member 5C

Gene	Function
KIFC1	Kinesin family member C1
LDB3	LIM domain binding 3
MAF	v-maf musculoaponeurotic fibrosarcoma oncogene homolog (avian)
MFSD7	major facilitator superfamily domain containing 7
MYO1G	myosin IG
NCAPH2	non-SMC condensin II complex, subunit H2
NEK11	NIMA (never in mitosis gene a)- related kinase 11
OR7E104P	olfactory receptor, family 7, subfamily E, member 104 pseudogene
PCDH17	protocadherin 17
PDZRN3	PDZ domain containing ring finger 3
PODNL1	podocan-like 1
PPFIA3	protein tyrosine phosphatase, receptor type, f polypeptide (PTPRF), interacting protein (liprin), alpha 3
PRF1	perforin 1 (pore forming protein)
PTCRA	pre T-cell antigen receptor alpha
PTGER1	Prostaglandin E receptor 1 (subtype EP1), 42kDa
RRP1	ribosomal RNA processing 1 homolog (S. cerevisiae)
SERP2	Stress-associated endoplasmic reticulum protein family member 2
SEZ6L	seizure related 6 homolog (mouse)-like
SGCD	sarcoglycan, delta (35kDa dystrophin-associated glycoprotein)
STAT5A	signal transducer and activator of transcription 5A
SYCE1L	synaptonemal complex central element protein 1-like
SYNPO2L	synaptopodin 2-like
THSD7A	thrombospondin, type I, domain containing 7A
TNFRSF10C	Tumor necrosis factor receptor superfamily, member 10c, decoy without an intracellular domain
TRPC3	transient receptor potential cation channel, subfamily C, member 3
TSNARE1	t-SNARE domain containing 1
VIL1	villin 1
VWCE	von Willebrand factor C and EGF domains
ZFAT	zinc finger and AT hook domain containing
ZFPM2	zinc finger protein, multitype 2
ZNF527	zinc finger protein 527
ZNF8	zinc finger protein 8
